# Supplementary material for: Gut content metabarcoding of specialized feeders is not a replacement for environmental DNA assays of seawater in reef environments
Source: PeerJ. 2023 Sep 27;11:e16075. doi: 10.7717/peerj.16075 (PMC10542274; doi:10.7717/peerj.16075)
Supplement: Supplemental Information 2 — The relationship of eukaryotic community assemblages identified in each sample using the Jaccard index for the factors “method” and “site” are shown, with different combinations indicated by shapes (method) and colours (site) in the legend. Since sites for seawater and C. lunulatus gut samples were not always the same, we reran the PCO comparing presence/absence of eukaryotic families detected based on (C) 18S and (D) ITS2 sequences only for sites where seawater and guts were sampled (DS4, DS6, and DS13). [file peerj-11-16075-s002.docx]

**S2 Appendix**. Principal Co-ordinate Analysis (PCO) comparing presence/absence of eukaryotic families detected based on (A) 18S and (B) ITS2 sequences isolated from seawater and oval butterflyfish (*Chaetodon lunulatus*) gut samples collected at 10 sites from Dongsha Atoll (Taiwan) in the South China Sea. The relationship of eukaryotic community assemblages identified in each sample using the Jaccard index for the factors “method” and “site” are shown, with different combinations indicated by shapes (method) and colours (site) in the legend. Since sites for seawater and *C. lunulatus* gut samples were not always the same, we reran the PCO comparing presence/absence of eukaryotic families detected based on (C) 18S and (D) ITS2 sequences only for sites where seawater *and* guts were sampled (DS4, DS6, and DS13).

(A)


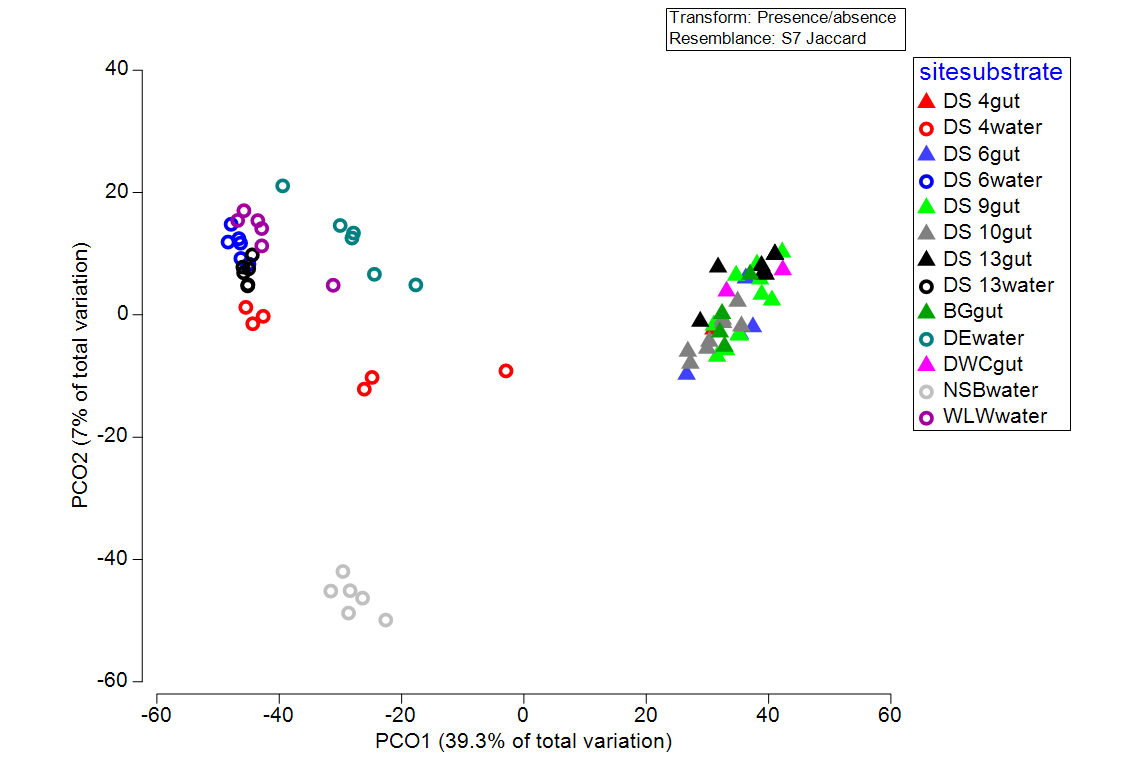


(b)


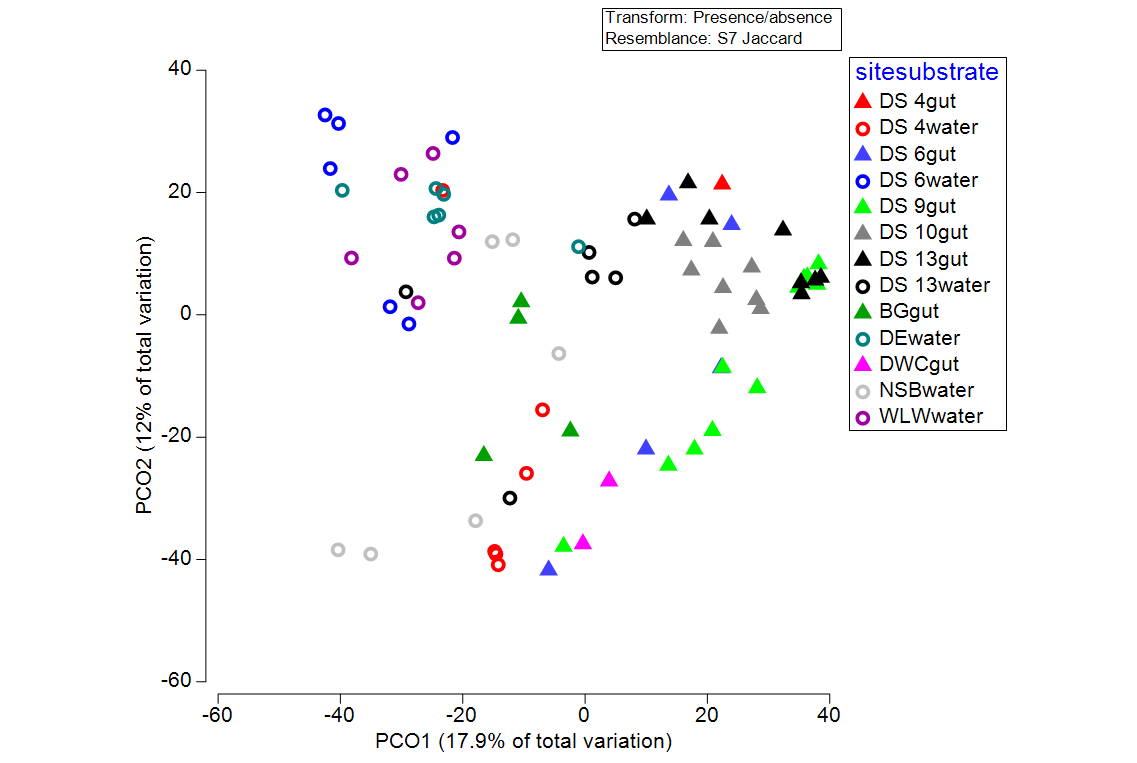


(c)


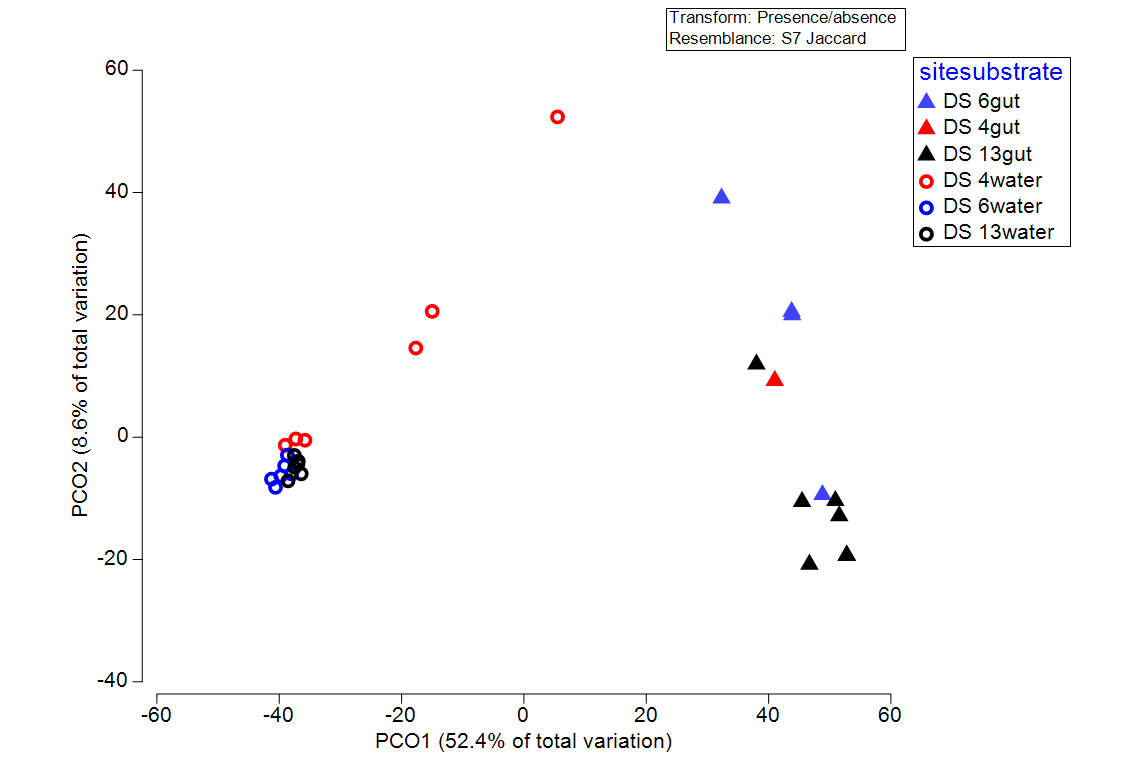


(d)

**
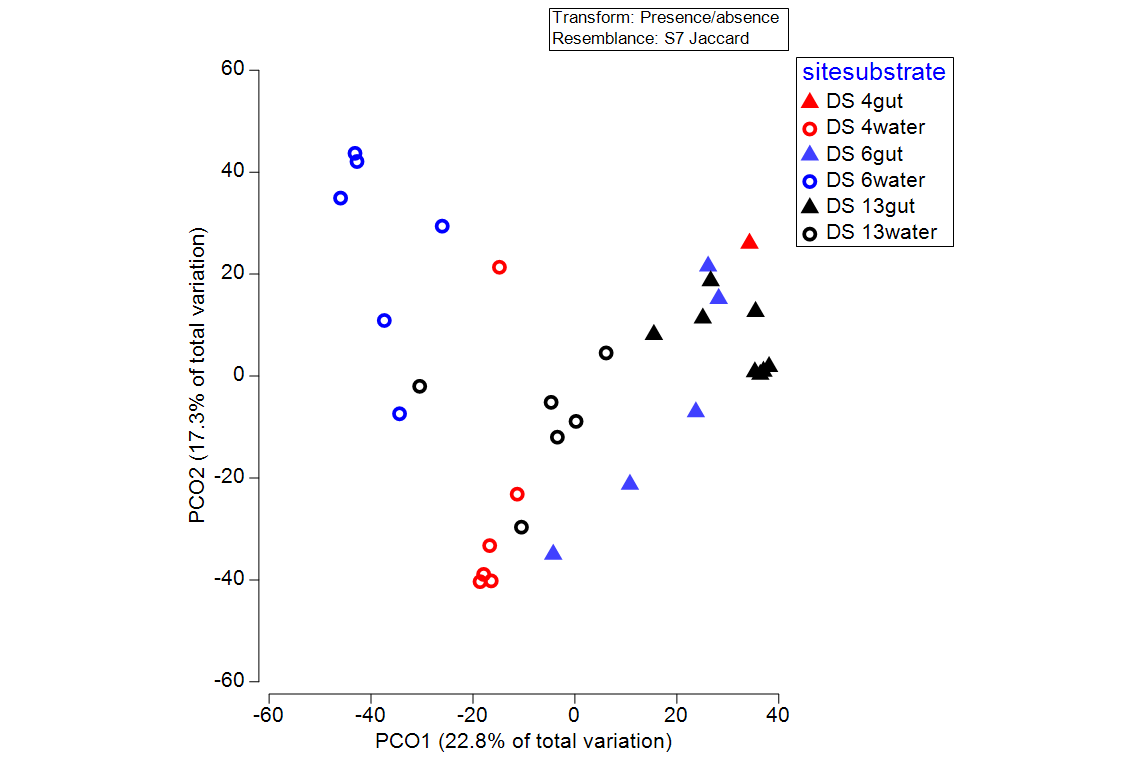
**
