# Supplementary material for: Gut content metabarcoding of specialized feeders is not a replacement for environmental DNA assays of seawater in reef environments
Source: PeerJ. 2023 Sep 27;11:e16075. doi: 10.7717/peerj.16075 (PMC10542274; doi:10.7717/peerj.16075)
Supplement: Supplemental Information 4 — Post quality filtered (QF) reads indicate those remaining following trimming for base quality (phred score ­ > 30) and minimum length (18S, 200 base pairs; ITS2, 100 base pairs) using BBDuk version 36.92 (Bushnell, 2017). [file peerj-11-16075-s004.docx]

**S4 Appendix**. 18Suni assay and ITS2 assay sequence information per replicate from seawater and oval individual butterflyfish (*Chaetodon lunulatus*) gut samples collected at 10 sites from Dongsha Atoll (Taiwan) in the South China Sea. Post quality filtered (QF) reads indicate those remaining following trimming for base quality (phred score ­> 30) and minimum length (18S, 200 base pairs; ITS2, 100 base pairs) using *BBDuk* version 36.92 (Bushnell 2017).

| Sample_ID | Location (Abbreviation) | Date | Latitude | Longitude | Number of 18S reads | Number of post-QF 18S reads | Number of ITS2 reads | Number of post-QF ITS2 reads |
| --- | --- | --- | --- | --- | --- | --- | --- | --- |
| Water sample |  |  |  |  |  |  |  |  |
| DGX1 | Dongsha Site 4 (DS 4) | 27/04/2018 | 20.63991 | 116.83894 | 92,107 | 87,051 | 55,737 | 55,183 |
| DGX2 | Dongsha Site 4 (DS 4) | 27/04/2018 | 20.63991 | 116.83894 | 85,963 | 81,702 | 62,261 | 49,994 |
| DGX3 | Dongsha Site 4 (DS 4) | 27/04/2018 | 20.63991 | 116.83894 | 96,563 | 92,624 | 43,755 | 43,135 |
| DGX4 | Dongsha Site 4 (DS 4) | 27/04/2018 | 20.63991 | 116.83894 | 181,647 | 174,186 | 69,105 | 68,239 |
| DGX5 | Dongsha Site 4 (DS 4) | 27/04/2018 | 20.63991 | 116.83894 | 25,972 | 21,768 | 49,721 | 49,012 |
| DGX6 | Dongsha Site 4 (DS 4) | 27/04/2018 | 20.63991 | 116.83894 | 22,664 | 19,719 | 18,767 | 18,143 |
| DGX7 | Dongsha Site 6 (DS 6) | 28/04/2018 | 20.70420 | 116.80710 | 94,472 | 88,259 | 59,157 | 58,249 |
| DGX8 | Dongsha Site 6 (DS 6) | 28/04/2018 | 20.70420 | 116.80710 | 98,054 | 92,299 | 39,574 | 38,505 |
| DGX9 | Dongsha Site 6 (DS 6) | 28/04/2018 | 20.70420 | 116.80710 | 153,385 | 144,435 | 41,038 | 39,777 |
| DGX10 | Dongsha Site 6 (DS 6) | 28/04/2018 | 20.70420 | 116.80710 | 129,719 | 124,257 | 48,138 | 47,768 |
| DGX11 | Dongsha Site 6 (DS 6) | 28/04/2018 | 20.70420 | 116.80710 | 52,248 | 47,400 | 45,871 | 44,882 |
| DGX12 | Dongsha Site 6 (DS 6) | 28/04/2018 | 20.70420 | 116.80710 | 124,029 | 120,362 | 56,710 | 55,857 |
| DGX13 | Dongsha Site 13 (DS 13) | 29/04/2018 | 20.70334 | 116.73715 | 181,285 | 165,207 | 112,725 | 111,225 |
| DGX14 | Dongsha Site 13 (DS 13) | 29/04/2018 | 20.70334 | 116.73715 | 45,847 | 42,979 | 50,525 | 49,988 |
| DGX15 | Dongsha Site 13 (DS 13) | 29/04/2018 | 20.70334 | 116.73715 | 72,342 | 66,276 | 59,969 | 59,285 |
| DGX16 | Dongsha Site 13 (DS 13) | 29/04/2018 | 20.70334 | 116.73715 | 65,529 | 63,152 | 105,052 | 103,642 |
| DGX17 | Dongsha Site 13 (DS 13) | 29/04/2018 | 20.70334 | 116.73715 | 82,586 | 78,260 | 39,498 | 39,223 |
| DGX18 | Dongsha Site 13 (DS 13) | 29/04/2018 | 20.70334 | 116.73715 | 73,784 | 63,399 | 32,108 | 31,607 |
| DGX19 | Dongsha East (DE) | 30/04/2018 | 20.70017 | 116.92455 | 111,157 | 92,140 | 37,311 | 37,010 |
| DGX20 | Dongsha East (DE) | 30/04/2018 | 20.70017 | 116.92455 | 78,820 | 75,543 | 38,967 | 38,693 |
| DGX21 | Dongsha East (DE) | 30/04/2018 | 20.70017 | 116.92455 | 106,468 | 98,244 | 40,789 | 39,964 |
| DGX22 | Dongsha East (DE) | 30/04/2018 | 20.70017 | 116.92455 | 219,331 | 210,403 | 3 | 3 |
| DGX23 | Dongsha East (DE) | 30/04/2018 | 20.70017 | 116.92455 | 188,963 | 168,224 | 65,267 | 64,329 |
| DGX24 | Dongsha East (DE) | 30/04/2018 | 20.70017 | 116.92455 | 74,221 | 71,199 | 68,932 | 68,066 |
| DGX25 | West Lagoon Wreck (WLW) | 01/05/2018 | 20.70625 | 116.70194 | 82,045 | 62,460 | 63,049 | 62,048 |
| DGX26 | West Lagoon Wreck (WLW) | 01/05/2018 | 20.70625 | 116.70194 | 62,402 | 58,776 | 27,441 | 27,021 |
| DGX27 | West Lagoon Wreck (WLW) | 01/05/2018 | 20.70625 | 116.70194 | 163,920 | 132,116 | 49,723 | 48,675 |
| DGX28 | West Lagoon Wreck (WLW) | 01/05/2018 | 20.70625 | 116.70194 | 93,459 | 80,693 | 40,066 | 39,267 |
| DGX29 | West Lagoon Wreck (WLW) | 01/05/2018 | 20.70625 | 116.70194 | 98,843 | 93,097 | 93,690 | 90,100 |
| DGX30 | West Lagoon Wreck (WLW) | 01/05/2018 | 20.70625 | 116.70194 | 62,092 | 56,430 | 46,568 | 45,546 |
| DGX31 | Northeast Seagrass Bed (NSB) | 02/05/2018 | 20.70518 | 116.72871 | 63,125 | 58,791 | 32,585 | 31,190 |
| DGX32 | Northeast Seagrass Bed (NSB) | 02/05/2018 | 20.70518 | 116.72871 | 76,569 | 69,960 | 140,095 | 138,364 |
| DGX33 | Northeast Seagrass Bed (NSB) | 02/05/2018 | 20.70518 | 116.72871 | 110,980 | 105,493 | 59,333 | 57,853 |
| DGX34 | Northeast Seagrass Bed (NSB) | 02/05/2018 | 20.70518 | 116.72871 | 69,142 | 66,728 | 62,560 | 60,747 |
| DGX35 | Northeast Seagrass Bed (NSB) | 02/05/2018 | 20.70518 | 116.72871 | 55,549 | 52,583 | 57,762 | 55,677 |
| DGX36 | Northeast Seagrass Bed (NSB) | 02/05/2018 | 20.70518 | 116.72871 | 86,725 | 80,234 | 53,349 | 50,622 |
|  |  |  |  |  |  |  |  |  |
| Gut sample |  |  |  |  |  |  |  |  |
| Clu1a | Dongsha Site 6 (DS 6) | 28/04/2018 | 20.70420 | 116.80710 | 345,957 | 296,432 | 49,190 | 47,614 |
| Clu2a | Dongsha Site 6 (DS 6) | 28/04/2018 | 20.70420 | 116.80710 | 32,736 | 28,011 | 70,717 | 67,928 |
| Clu3a | Dongsha Site 6 (DS 6) | 28/04/2018 | 20.70420 | 116.80710 | 47,853 | 43,086 | 57,228 | 55,474 |
| Clu4a | Dongsha Site 6 (DS 6) | 28/04/2018 | 20.70420 | 116.80710 | 23,393 | 15,571 | 39,134 | 38,293 |
| Clu5a | Dongsha Site 6 (DS 6) | 28/04/2018 | 20.70420 | 116.80710 | 35,816 | 30,427 | 89,818 | 87,552 |
| Clu6a | Dongsha Site 4 (DS 4) | 27/04/2018 | 20.63991 | 116.83894 | 28,535 | 27,484 | 60,021 | 58,589 |
| Clu7a | Dongsha Site 9 (DS 9) | 01/05/2018 | 20.74059 | 116.78586 | 202,733 | 152,866 | 58,059 | 56,985 |
| Clu8a | Dongsha Site 9 (DS 9) | 01/05/2018 | 20.74059 | 116.78586 | 23,725 | 21,912 | 58,111 | 57,253 |
| Clu9a | Dongsha Site 9 (DS 9) | 01/05/2018 | 20.74059 | 116.78586 | 115,948 | 82,081 | 49,815 | 48,904 |
| Clu10a | Dongsha Site 9 (DS 9) | 01/05/2018 | 20.74059 | 116.78586 | 192,913 | 171,794 | 77,153 | 75,631 |
| Clu11a | Dongsha Site 9 (DS 9) | 01/05/2018 | 20.74059 | 116.78586 | 28,080 | 24,214 | 47,674 | 47,099 |
| Clu12a | Dongsha Site 9 (DS 9) | 01/05/2018 | 20.74059 | 116.78586 | 26,920 | 25,434 | 44,168 | 43,717 |
| Clu13a | Dongsha Site 9 (DS 9) | 01/05/2018 | 20.74059 | 116.78586 | 23,547 | 22,093 | 49,947 | 49,518 |
| Clu14a | Dongsha West Channel (DWC) | 28/04/2018 | 20.64979 | 116.69519 | 36,957 | 33,384 | 56,030 | 54,611 |
| Clu15a | Dongsha West Channel (DWC) | 28/04/2018 | 20.64979 | 116.69519 | 135,776 | 103,420 | 53,029 | 50,731 |
| Clu16a | Dongsha Site 9 (DS 9) | 01/05/2018 | 20.74059 | 116.78586 | 28,797 | 27,257 | 59,220 | 58,955 |
| Clu17a | Dongsha Site 9 (DS 9) | 01/05/2018 | 20.74059 | 116.78586 | 28,289 | 25,081 | 49,435 | 49,084 |
| Clu18a | Dongsha Site 9 (DS 9) | 01/05/2018 | 20.74059 | 116.78586 | 38,165 | 36,095 | 51,446 | 50,611 |
| Clu19a | Dongsha Site 9 (DS 9) | 01/05/2018 | 20.74059 | 116.78586 | 27,495 | 24,555 | 39,028 | 38,616 |
| Clu20a | Dongsha Site 13 (DS 13) | 29/04/2018 | 20.70334 | 116.73715 | 27,004 | 23,762 | 74,228 | 73,330 |
| Clu21a | Dongsha Site 13 (DS 13) | 29/04/2018 | 20.70334 | 116.73715 | 125,395 | 113,529 | 51,793 | 51,047 |
| Clu22a | Dongsha Site 13 (DS 13) | 29/04/2018 | 20.70334 | 116.73715 | n/a | n/a | 45,171 | 44,360 |
| Clu23a | Dongsha Site 13 (DS 13) | 29/04/2018 | 20.70334 | 116.73715 | 45,564 | 42,826 | 41,047 | 40,668 |
| Clu24a | Dongsha Site 13 (DS 13) | 29/04/2018 | 20.70334 | 116.73715 | 178,940 | 166,658 | 44,138 | 43,539 |
| Clu25a | Dongsha Site 13 (DS 13) | 29/04/2018 | 20.70334 | 116.73715 | 149,769 | 138,042 | 51,520 | 51,000 |
| Clu26a | Dongsha Site 13 (DS 13) | 29/04/2018 | 20.70334 | 116.73715 | 223,957 | 191,048 | 36,912 | 36,437 |
| Clu27a | Dongsha Site 13 (DS 13) | 29/04/2018 | 20.70334 | 116.73715 | 189,510 | 179,579 | 33,056 | 32,616 |
| Clu28b | Dongsha Site 10 (DS 10) | 29/04/2018 | 20.66452 | 116.77552 | 194,560 | 176,458 | 39,800 | 39,530 |
| Clu29b | Dongsha Site 10 (DS 10) | 29/04/2018 | 20.66452 | 116.77552 | 104,458 | 76,504 | 62,373 | 61,671 |
| Clu30b | Dongsha Site 10 (DS 10) | 29/04/2018 | 20.66452 | 116.77552 | 109,077 | 103,321 | 45,845 | 45,315 |
| Clu31b | Dongsha Site 10 (DS 10) | 29/04/2018 | 20.66452 | 116.77552 | n/a | n/a | n/a | n/a |
| Clu32b | Dongsha Site 10 (DS 10) | 29/04/2018 | 20.66452 | 116.77552 | 132,859 | 125,199 | 26,260 | 26,041 |
| Clu33b | Dongsha Site 10 (DS 10) | 29/04/2018 | 20.66452 | 116.77552 | 28,624 | 25,720 | 26,854 | 26,630 |
| Clu34b | Dongsha Site 10 (DS 10) | 29/04/2018 | 20.66452 | 116.77552 | 122,594 | 115,125 | 34,686 | 34,447 |
| Clu35b | Dongsha Site 10 (DS 10) | 29/04/2018 | 20.66452 | 116.77552 | 28,289 | 25,876 | 31,679 | 31,199 |
| Clu36b | Dongsha Site 10 (DS 10) | 29/04/2018 | 20.66452 | 116.77552 | 163,173 | 152,419 | 39,944 | 39,487 |
| Clu37b | Boo’s Groove (BG) | 30/04/2018 | 20.77151 | 116.76759 | 122,578 | 103,180 | 53,681 | 51,988 |
| Clu38b | Boo’s Groove (BG) | 30/04/2018 | 20.77151 | 116.76759 | 57,157 | 47,128 | 46,002 | 44,915 |
| Clu39b | Boo’s Groove (BG) | 30/04/2018 | 20.77151 | 116.76759 | 44,270 | 37,596 | 54,222 | 53,519 |
| Clu40b | Boo’s Groove (BG) | 30/04/2018 | 20.77151 | 116.76759 | 27,169 | 23,770 | 64,643 | 63,152 |

Reference:

Bushnell, B (2017) BBDuk Trimmer. Available at: <http://jgi.doe.gov/data-and-tools/bb-tools/>
