# Supplementary material for: Gut content metabarcoding of specialized feeders is not a replacement for environmental DNA assays of seawater in reef environments
Source: PeerJ. 2023 Sep 27;11:e16075. doi: 10.7717/peerj.16075 (PMC10542274; doi:10.7717/peerj.16075)
Supplement: Supplemental Information 5 — P-values in bold are significant at p < 0.05. MC (Monte Carlo) tests were used in the case of low sample permutations and are indicated in italics. [file peerj-11-16075-s005.docx]

**S5 Appendix**. 18Suni assay and ITS2 assay pairwise comparisons in PERMANOVA. P-values in bold are significant at *p* < 0.05. MC (Monte Carlo) tests were used in the case of low sample permutations and are indicated in italics.

| 18Suni assay | | | | | | |
| --- | --- | --- | --- | --- | --- | --- |
| Seawater | | | Gut content | | |  |
| Sites | t | *p* | Sites | t | *p* | MC |
| DS4 - DS6 | 1.974 | **0.002** | DS4 - DS6 | 1.131 | 0.318 | *MC* |
| DS4 - DS13 | 1.953 | **0.002** | DS4 - DS9 | 1.405 | 0.085 | *MC* |
| DS4 - DE | 1.965 | **0.002** | DS4 - DS10 | 1.139 | 0.278 | *MC* |
| DS4 - NSB | 2.292 | **0.002** | DS4 - DS13 | 1.471 | 0.108 | *MC* |
| DS4 - WLW | 1.974 | **0.001** | DS4 - BG | 1.691 | 0.108 | *MC* |
| DS6 - DS13 | 3.378 | **0.002** | DS4 - DWC | 1.423 | 0.339 | *MC* |
| DS6 - DE | 2.763 | **0.002** | DS6 - DS9 | 1.163 | 0.200 |  |
| DS6 - NSB | 4.186 | **0.003** | DS6 - DS10 | 1.487 | **0.024** |  |
| DS6 - WLW | 2.767 | **0.002** | DS6 - DS13 | 1.304 | 0.118 |  |
| DS13 - DE | 2.725 | **0.002** | DS6 - BG | 1.805 | 0.**009** |  |
| DS13 - NSB | 3.785 | **0.002** | DS6 - DWC | 0.988 | 0.424 | *MC* |
| DS13 - WLW | 2.328 | **0.003** | DS9 - DS10 | 1.862 | **0.001** |  |
| DE - NSB | 2.911 | **0.002** | DS9 - DS13 | 1.673 | **0.008** |  |
| WLW - NSB | 3.274 | **0.003** | DS9 - BG | 1.772 | **0.002** |  |
| DE - WLW | 1.944 | **0.005** | DS9 - DWC | 1.266 | 0.152 | *MC* |
|  |  |  | DS10 - DS13 | 2.254 | **0.001** |  |
|  |  |  | DS10 - BG | 1.731 | **0.008** |  |
|  |  |  | DS10 - DWC | 1.466 | 0.076 | *MC* |
|  |  |  | DS13 - BG | 2.177 | **0.007** |  |
|  |  |  | DS13 - DWC | 0.838 | 0.590 | *MC* |
|  |  |  | BG - DWC | 1.637 | 0.087 | *MC* |

| ITS2 assay | | | | | |  |
| --- | --- | --- | --- | --- | --- | --- |
| Seawater | | | Gut content | | |  |
| Sites | t | *p* | Sites | t | *p* | MC |
| DS4 - DS6 | 2.0577 | **0.004** | DS6 - DS4 | 1.0749 | 0.350 | *MC* |
| DS4 - DS13 | 1.7800 | **0.004** | DS6 - DS9 | 1.6381 | **0.016** |  |
| DS4 - DE | 1.5971 | **0.018** | DS6 - DWC | 1.3816 | 0.153 | *MC* |
| DS4 - NSB | 1.3203 | 0.058 | DS6 - DS13 | 1.9669 | **0.001** |  |
| DS4 - WLW | 2.3756 | **0.002** | DS6 - DS10 | 2.1578 | **0.001** |  |
| DS6 - DS13 | 2.2184 | **0.003** | DS6 - BG | 1.7049 | **0.008** |  |
| DS6 - DE | 1.4868 | **0.006** | DS4 - DS9 | 1.7032 | **0.041** | *MC* |
| DS6 - NSB | 1.9594 | **0.002** | DS4 - DWC | 2.8956 | 0.150 | *MC* |
| DS6 - WLW | 2.3104 | **0.002** | DS4 - DS13 | 1.4471 | 0.101 | *MC* |
| DS13 - DE | 1.6496 | **0.007** | DS4 - DS10 | 2.1121 | **0.010** | *MC* |
| DS13 - NSB | 2.0556 | **0.002** | DS4 - BG | 1.4660 | 0.147 | *MC* |
| DS13 - WLW | 2.5170 | **0.002** | DS9 - DWC | 1.8729 | 0.019 | *MC* |
| DE - NSB | 1.5133 | **0.002** | DS9 - DS13 | 2.0129 | **0.001** |  |
| DE - WLW | 1.6898 | **0.003** | DS9 - DS10 | 2.6052 | **0.001** |  |
| NSB - WLW | 2.1836 | **0.003** | DS9 - BG | 2.4668 | **0.001** |  |
|  |  |  | DWC - DS13 | 2.1887 | **0.005** | *MC* |
|  |  |  | DWC - DS10 | 2.2113 | **0.005** | *MC* |
|  |  |  | DWC - BG | 1.2260 | 0.242 | *MC* |
|  |  |  | DS13 - DS10 | 2.1896 | **0.001** |  |
|  |  |  | DS13 - BG | 2.4717 | **0.002** |  |
|  |  |  | DS10 - BG | 2.4151 | **0.002** |  |
